# Digital technology and practices for school improvement: innovative digital school model

**DOI:** 10.1186/s41039-018-0094-8

**Published:** 2018-12-28

**Authors:** Liisa Ilomäki, Minna Lakkala

**Affiliations:** Faculty of Educational Sciences, University of Helsinki, P.O. Box 9, 00014 Helsinki, Finland

**Keywords:** Lower secondary, School, School improvement, Innovation, Digital technology

## Abstract

The aim of this study was to create a model which describes the main elements for improving schools with digital technology and helps to reveal differences between schools and identify their best practices and challenges. The innovative digital school model (IDI school) offers a framework for research but also a research-based model for schools to examine their own practices with digital technologies. The model combines previous research on school improvement, creation of innovations, and digital technology in education as a special case of innovations and learning as knowledge creation to define six main elements describing an innovative, digital school: visions of the school, leadership, practices of the teaching community, pedagogical practices, school-level knowledge practices and digital resources. The model was applied to investigate three basic education schools. The results indicate that the model worked: we found essential differences between the schools and their best practices and challenges for improvement. It worked particularly well for those elements, which are mainly the responsibility for leadership inside a school. The differences of various elements between schools were not based on socioeconomic background but on the school-level practices. As a conclusion, we suggest that to improve schools with digital technology, all elements of the model should be included in the evaluation and development process.

## Introduction

In today’s world, education is facing major challenges: it is expected to provide children and teenagers with competencies they will need in the future, to consider informal ways of learning, and to apply digital technologies and modern pedagogical methods to answer these challenges (EU, 2010). However, schools have not managed to meet all these challenges: e.g. digital technology has not yet been applied much in education, although it is widely in use elsewhere in the society and in work life (EU 2013; Livingstone 2012); students do not acquire sufficient competence at school to undertake university studies (such as collaboration, planning, independent learning, digital competence or working with knowledge) (Hautamäki et al. 2012; Kiili 2012; Lundahl et al. 2010); and there are major differences between countries and schools in reaching these skill levels (such as problem-solving skills, OECD 2014, 2017). There have been promising results that some pedagogical practices related to student centredness, real-life activities and group work have increased at schools between 2001 and 2011. Such pedagogical practices are often linked to the use of digital technology (OECD 2014).

There is a large body of research about using digital technology in schools, in classrooms and among teachers and students, but often these studies concentrate on only one or two phenomena of education and technology (e.g. classroom cases, or technical competence of teachers and students), thus isolating the object of study from the broader context of a school. Unless a more comprehensive view is adopted in the efforts of developing a school, there is little chance of innovation programmes having any lasting effect (Wikeley et al. 2005). Wong and Li (2011) investigated the connection between information and communication technology (ICT) implementation and pedagogical change. They concluded that organisational interventions and pedagogical interventions interacted with each other in effecting changes in student learning. Korhonen et al. (2014) introduced an innovative school community model, which addresses the development of four elements: students’ learning and learning environments, teachers’ professionalism, leadership and partnerships, as central to the advancement of educational innovation related to versatile use of digital technology. The model is generic, which leaves considerable room for interpretation in examining how current practices in a school should be evaluated and improved.

To investigate schools, we followed the *sociocultural approach to learning* (John-Steiner and Mann 1996; Packer and Goicoechea 2000): a school is an environment of collaborative, social activities of teachers, pupils and other participants; and their activities shape and transform its culture, values, practices and other specific characteristics. This approach also has an impact on our methodological choices: we mainly investigated *practices* rather than *beliefs or thoughts*.

The interest in the present study is in exploring the critical elements to be considered and the development processes needed in schools for reforming school education. Our specific focus is on the use of digital technology: how new digital technology has been applied and how it could be used to improve pedagogical and knowledge practices.

*School* is a complicated object to study: it consists of *various administrative levels*, from the national policy level to classrooms; *various actors*, such as school staff and pupils inside a school as well as parents and local school administrators outside a school; *contradictory aims*, such as aiming to ensure relevant competence levels for pupils in the future, but simultaneously, carrying on the traditions and history of society. For the complexity of a school as a research object, the theoretical background for the present study is multifaceted: research about school improvement, research about innovation, research about pedagogical practices (especially the collaborative knowledge creation traditions) and studies about digital technologies in education.

The connection to societal goals is essential for a school; it forms the external structure and resources for schools—which certainly have a strong impact (e.g. Ranson et al. 2005)—but the responsibility for improving an individual school from the *inside* rests with the principal and the teachers. For this reason, the focus in the present study is on the elements and practices inside individual schools, bearing in mind the external factors and stakeholders. The reason for leaving the external administration outside the approach of the study is pragmatic: we want to create a model for schools for their own use, to reflect and improve those practices that they are able to change themselves. An individual school can seldom affect upper-level administrative decisions, but schools always possess some autonomy to make changes in the work of teachers and pupils. As Lemke (2001) emphasised, educational researchers should be explicit about the level of phenomena and the primary unit of analysis that the investigation is focusing on, but also be aware of the influence of the phenomena at upper and lower levels (e.g. municipal-level administrative decisions or individual teachers’ personal motives). Leclerc et al. (2012) investigated individual principals and teachers and made school-level conclusions based on these data. This was similar to work by Peck et al. (2009) when they were investigating innovations in schools. The present study focuses on classroom and school-level practices by interviewing individuals (teachers and principals), observing teaching practices and by conducting surveys for teachers and pupils. We presuppose that there is a strong and essential interaction between the different levels; this is a major starting point of our study.

In the following section, we first describe how the study relates to previous research approaches and then introduce the innovative digital school (IDI school) model: its basic elements and their connection with previous research. The framework has been applied in our study to examine schools. In the empirical section, the application of the model has been examined through case studies from three comprehensive schools.

## Review of relevant previous research approaches for developing the model

### Research on school improvement and change

School improvement is aimed at improving student outcomes, wherever the change takes place (Creemers and Reezigt 2005). The large body of research about school improvement is one of the cornerstones of understanding the structures and practices of schools, such as leadership practices, teachers’ professional collaboration or pedagogical practices. Studies about school improvement have indicated how schools have benefited from restructuring their common practices, such as teachers’ tasks, activities and learning practices, leadership practices and the ways pedagogical methods are organised, in order to meet the developmental challenges (Crook et al. 2010; Harris 2002b; OECD 2015). The elements of consensus about the vision (in vision of the school) and shared leadership (in leadership) are based on the studies presented here.

The school improvement movement and related research are strongly connected to educational systems and the policy-based and societal goals of education. Countries differ in their goals and views about school improvement, and the means for improving education can even be contradictory—leading also to quite different results (Hargreaves 2011; OECD 2014 2015). In countries such as the UK, the approach has been hierarchical top-down, whereas in the Nordic countries, the emphasis is on democracy, meaning the goal is to give schools and teachers responsibility for the improvement (Sahlberg 2011; Wrigley 2003). The elements of practices of the teaching society are based on the approach of teachers’ responsibility for the school improvement.

Researchers have defined some necessary characteristics for a school as a learning organisation (Senge et al. 1994). These are mutual trust and willingness to engage in open communication by the participants (Creemers and Reezigt 2005; Harris 2002b; Leclerc et al. 2012; Senge et al. 1994); teachers’ shared values and visions, which focus on student learning (Leclerc et al. 2012); and collaborative knowledge-sharing as a tool for continuous growth of both teachers and schools. Knowledge sharing is a fundamental transformation of the teaching profession itself and is a route for creating collaborative cultures (Fullan 2001; Leclerc et al. 2012; Pedder and MacBeath 2008). Furthermore, staff members have opportunities to influence the school’s activities and policies (Harris 2002b; Newmann et al. 2000), teacher collaboration is further supported by practical arrangements such as allocating time for teacher collaboration and teachers assume collective responsibility for attaining goals (Creemers and Reezigt 2005; Leclerc et al. 2012; Newmann et al. 2000). The elements of practices of the teaching community and school-level knowledge practices are based on the studies presented here.

For school improvement, the role of the school principal is essential. The principal manages the processes, motivates, organises and involves the staff in improvement, shares values for creating and supporting common visions (DuFour and Mattos 2013; Harris 2002a) and understands teachers’ learning as a vehicle for the school’s continuous improvement (Earley 2010). Leadership affects the atmosphere for collaboration and experimentation (Wong and Li 2011). School leadership is best understood as a distributed practice, stretched over the school’s social and situational contexts, which is also beneficial for teachers (Facer 2012; OECD 2015; Spillane et al. 2004). It is an interactive process to build social capacity and trust, and to support networking (Harris 2002a; Leclerc et al. 2012; Resnick and Spillane 2006). A challenge for a principal as an educational leader is the requirement for networking with other principals, administrators and other external stakeholders, which provides new perspectives and promotes the creation of effective and sustainable improvement (Hargreaves and Fink 2003; Harris 2010). The elements of leadership are based on the studies presented here.

In addition to research on school improvement, the research on knowledge work gives essential inspiration on how to view schools as organisations. Brown and Duguid (2001) emphasised practices and their travelling within an organisation and through sub-cultures. This sharing and collaborative creation of knowledge and practices is realised via boundary objects, such as common ways of working or shared objects to be developed. Brown and Duguid were investigating business firms, but schools are also knowledge work organisations. The elements of development practices (in practices of the teaching community) and common knowledge practices with technology (in school-level knowledge practices) are based on the ideas of Brown and Duguid.

### Research on innovation applied in school context

Research concerning innovation provides essential added value to understanding the improvement of pedagogical practices. There are various definitions of innovation, differing between the level of focus and the novelty of the innovation (OECD 2010, 2014). Some definitions regard only fundamentally new change as innovation, some also accept inclusion of issues that are novel in the context of the users. Messmann and Mulder (2011) defined an innovation as follows: ‘products or processes that are new and applicable for a certain individual, group or organisation and that are useful for the same or a different individual, group or organization’ (p. 66). This definition is close to the approach adopted in the present study. The emergence, acceptance and distribution of innovations that focus on the connection between individuals and organisations are especially important when answering the question about how educational innovations are adopted and what are the conditions for their dissemination.

An educational innovation succeeds or fails with the teachers who shape it (Lieberman and Pointer Mace 2008). In every significant change, the locus of innovations in practice could be traced to insights and initiatives of individuals, and collective negotiations and actions through which the changes have been achieved (Peck et al. 2009). Messmann and Mulder (2011) found in their study that the most powerful processes of learning and innovation took place in informal professional and personal relationships and in teachers’ communities. Teachers were motivated to work for change, and their positive individual image was framed by the experience of social support by colleagues and the supervisor as well as a stimulating climate for innovation. This also created a social norm that innovative work was appreciated. Several matters facilitated innovative work behaviour: competence, impact, responsibility for change, motivation for change, supervisor’s support, participative safety, supportive atmosphere and job complexity (see also Kunnari and Ilomäki 2016). Furthermore, in studies of teachers’ learning in innovation projects, experiments in practice and teacher learning go hand in hand (Bakkenes et al. 2010; Ilomäki et al. 2017). According to Bakkenes et al. (2010), informal learning brought fewer positive results than organised learning, especially reciprocal working with a peer or in a collaborative project team. Pedder and MacBeath (2008) argued that for schools (in the UK), the challenge appears to be in reasserting the values of learning, risk-taking, critical introspection, experimentation and innovation at all levels of the school organisation, and putting these into practice. Preconditions for innovation in organisations resemble the characteristics of learning communities: supporting teachers’ competence, autonomy and collegiality motivate teachers to change their teaching approaches (Lam et al. 2010; OECD 2015).

The elements of vision of the school and pedagogical collaboration and sharing of expertise and development practices (in practices of the teaching community) are based on the studies presented here.

#### Technology adoption as an innovation in school

The expectations about rapid acceptance and implementation of digital technology into educational practices have not been fulfilled (EU 2013), although some promising results indicate the connection between new pedagogical practices (= less teacher-centred) and the use of digital technology (Donnelly et al. 2011; Overbay et al. 2010; OECD 2014). In schools, technology is often still used for prevailing teaching methods, such as information sharing, or doing simple exercises, rather than for promoting collaborative or creative activities, solving complex problems or improving students’ digital competence (Livingstone 2012; OECD 2010).

Two alternative explanations for transforming educational practices associated with ICT have been suggested (Cuban et al. 2001; Twining et al. 2013): The first is a ‘slow revolution’ and support for existing practices, in which small changes accumulate over time and create a slow-motion transformation towards new ways of working. Only routines are replaced, and no changes are made in learning content or pedagogical practices. This explanation is anchored to the notion of a time lag between the invention of new technology, the adoption of innovations and the slow spread of its virtues through the general population. According to this explanation, the adoption of technology is an inevitable result which will come about anyway. The second explanation, ‘active transformation’ tries to account for the sustaining of teacher-centred practices: teachers and school make plans and decide how technology should be implemented in how best to answer to the specific challenges the school has. The curriculum content and/or processes will be changed, and these are changes that could not have taken place without digital technology.

There is a large body of studies about how digital technology has been implemented in education; e.g. what resources schools, teachers and students have; how much digital technology is used in classrooms; and what practices digital technology is used for (OECD 2010, 2011, 2014, 2015). First, it is essential that teachers and students have the opportunity to learn to use digital technology, and second, that they have meaningful and necessary resources to use it. Teachers’ digital competence, related to pedagogical understanding of using technology in education, is the corner stone of supporting students’ digital competence (Hakkarainen et al. 2000, 2001). The elements of pedagogical practices and digital resources are based on the studies presented here.

### Research on learning as knowledge creation

Those theoretical approaches emphasising learning as *collaborative knowledge creation* (Bereiter 2002; Paavola and Hakkarainen 2005; Hong and Sullivan 2009) have strongly influenced our views concerning the pedagogical development in schools through digital technologies. According to these approaches, teaching should primarily promote knowledge innovation and collective advancement of shared knowledge products (Scardamalia and Bereiter 2006; Hong and Sullivan 2009). Arguments for these approaches are the requirement to promote adaptive expertise, collaboration skills and capabilities to work creatively with knowledge, which are the competencies needed in education, working life and society in general. Recent discussions concerning the learning of ‘21st Century Skills’ have similarities with these ideas: school learning should focus more on supporting the development of the relevant competencies that are needed to cope with the challenges of the unknown future, instead of concentrating on content learning and routine tasks (Ananiadou and Claro 2009; Bell 2010).

Features of pedagogical practices representing the collaborative knowledge creation approach include learners’ engagement, goal-oriented production of knowledge objects for relevant purpose, collective efforts and resources and versatile use of modern technologies (Robin 2008; Bell 2010; Scardamalia and Bereiter 2006; Tan and McWilliam 2009). The role of technological applications in such practices is often to provide flexible tools for communication and networking, co-authoring of shared knowledge products and managing joint working processes (Lakkala et al. 2009). The elements of pedagogical practices are based on the studies presented in the two previous chapters.

Scardamalia and Bereiter (1999) suggested that to help students to succeed in the knowledge society, schools should become knowledge-building organisations, in which students are members, not clients. Their suggestions are in line with the ideas of learning as knowledge creation (in which tradition they have a profound contribution). The element of pupils’ involvement (in school-level knowledge practices) is based on the this approach.

### The elements of innovative digital school

Based on previous research approaches reviewed above and our own studies (Ilomäki and Lakkala 2011; Lakkala and Ilomäki 2013), we created the innovative digital school (IDI school) model for investigating whether schools use digital technology in an innovative way to improve pedagogical and working practices. In developing the model, we have emphasised leaning on relevant previous research approaches to avoid criticisms about creating a model based on occasional empirical findings, which leads to a quasi-theoretical model (Wikeley et al. 2005). However, we have also used a data-driven approach with extensive data from everyday practices of schools in order to avoid the gap between the theoretical model and ordinary practices in the field. Such data-driven elements, also acknowledged somewhat by research, are especially elements in school-level practices: physical premises (Cleveland and Fisher 2014; Gislason 2010) and pupils’ involvement in school level activities (Katsenou et al. 2015; Svanbjörnsdóttir et al. 2016). Table 1 presents the relationship between the elements of IDI School model with relevant research approaches, the main conclusions of previous studies related to the elements of our model and the main references.

**Table 1:** Elements of the IDI school model and their relationship with previous research approaches

Element	Research approach	The main conclusion	References
Vision of the school			
Visions of using digital technology	Research on technology as adoption of an innovation in school; research on school improvement and change	A shared vision is needed for continuous school improvement.	Cuban et al. (2001); Twining et al. (2013)
Consensus about the vision	Research on school improvement and change	A consensus of the vision enables collaboration directed to a same goal.	Leclerc et al. (2012)
Intentional development orientation	Research on innovation applied in school context	Intentional orientation is one of the corner stones for innovations.	Creemers and Reezigt (2005); Leclerc et al. (2012); OECD (2015); Rogers (2003)
Leadership			
Shared leadership	Research on school improvement and change	Shared leadership supports teachers’ participation and engagement in school-level activities by sharing the responsibility to several members of the community.	Facer (2012); Harris (2002a); Leclerc et al. (2012); OECD (2015); Spillane et al. (2004); Resnick and Spillane (2006)
Principal’s networking	Research on school improvement and change	Networking provides new perspectives and in this way, promotes the creation of improvements.	Hargreaves and Fink (2003); Harris (2010).
The role of the principal	Research on school improvement and change	Principal’s role is to manage, motivate, organise and involve the staff in atmosphere for collaboration and experimentation.	DuFour and Mattos (2013); Earley (2010); Harris (2002a); Rogers (2003); Wong and Li (2011)
Practices of the teaching community			
Pedagogical collaboration and sharing of expertise	Research on school improvement and change	Pedagogical collaboration and sharing supports teachers’ professional development as well as collaborative improvement of pedagogical practices	Fullan (2001); Harris (2002b); Leclerc et al. (2012); Pedder and MacBeath (2008)
Development practices	Research on school improvement and change; research on innovation applied in school context	Teachers’ development practices are an effective way to improve pupils learning and a way to improve teacher expertise.	Bakkenes et al. (2010); Harris (2002b); Messmann and Mulder (2011); Rogers (2003)
Networking of teachers	Research on school improvement and change	Networking opens the isolated teacher profession to new ideas and thinking. It is necessary for innovations.	Chapman (2008); Scimeca et al. (2009)
Pedagogical practices			
Perceptions of using digital technology in education	Research on technology as adoption of an innovation in school; research on learning as knowledge creation	Teachers’ perceptions of using technology affects the ways teachers use it with pupils. Perceptions are often more ‘advanced’ than the actual practices.	Bereiter (2002); Donnelly et al. (2011); Hakkarainen et al. (2001); Hong and Sullivan (2009); Scardamalia and Bereiter (2006)
Pedagogical practices with digital technology	Research on technology as adoption of an innovation in school; research on learning as knowledge creation	Pedagogical practices with technology should focus on complex issues and activities like knowledge creation and problem solving in order to advance pupils’ general competencies.	Bell (2010); Donnelly et al. (2011); Hakkarainen et al. (2001); OECD (2014)
School-level knowledge practices			
Common knowledge practices with technology	Research on learning as knowledge creation; research on knowledge work organisations	Common knowledge practices support learning and development in an organisation; in school, common practices help teachers and pupils because they give ‘standard’ models and ways of working.	Brown and Duguid (2001); Scardamalia and Bereiter (1999)
Physical premises	Data on previous phases of the model, research on learning environments	The school has sufficient and flexible premises for various pedagogical use	Cleveland and Fisher (2014); Gislason (2010)
Pupils’ involvement in school level activities	Research on learning as knowledge creation; action research tradition; data on previous phases of the model	Students are active members in the school community, not only as ‘objects of teaching’.	Katsenou et al. 2015; Scardamalia and Bereiter (1999) Svanbjörnsdóttir, Macdonald and Frímannsson (2016)
School-level networking	Research on technology as adoption of an innovation in school; research on school improvement and change	A networking school opens out to the society and thus receives new kinds of collaboration and learning opportunities for pupils and teachers.	Brown and Duguid (2001); Chapman (2008); Scimeca et al. (2009)
Digital resources			
Utility of technical resources	Research on technology as adoption of an innovation in school	The school has resources for teaching and learning with digital technology; and the resources are organised meaningful way helping teachers and pupils in using technology.	OECD (2014); Wong and Li (2011)
Pupils’ digital competence	Research on technology as adoption of an innovation in school	Pupils’ digital competence is acknowledged at school; pupils use technology in multiple ways, also at school and for school work. Learning digital technology in school ensures relevant competence for further education.	OECD (2010, 2011, 2014)
Teachers’ digital competence	Research on technology as adoption of an innovation in school	Teachers’ digital competence is sufficient for carrying out pedagogical practices with technology; they can also support pupils’ evolving digital competence.	OECD, 2010
Pedagogical and technical training and support	Research on technology as adoption of an innovation in school	Teachers get various kind of pedagogical and technical training and support at local and school level. In this way, teachers can improve their professional competence.	Hakkarainen et al. 2001

**Fig. 1:**
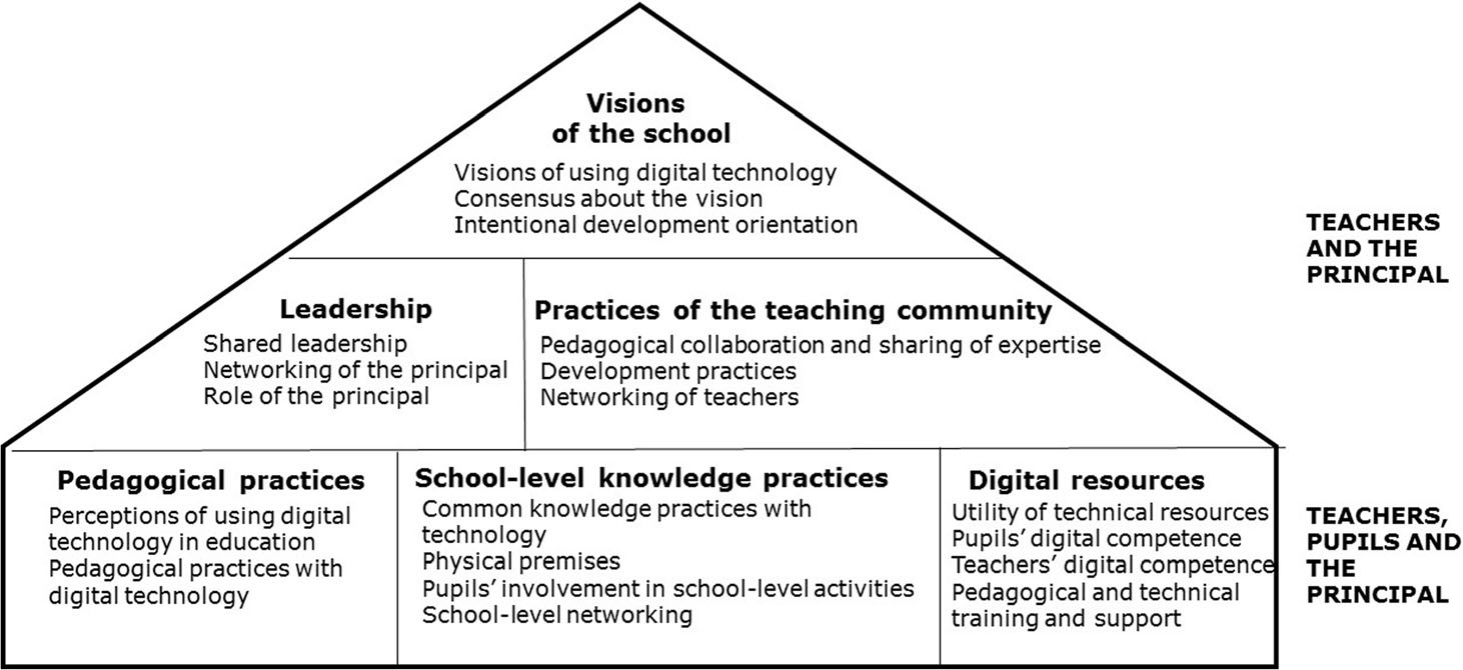
The innovative digital school model: elements of a school regarded as relevant for developing schools through digital technology

The elements are presented in visual form in Fig. 1.

## Aims and research questions of the study

The reason for developing the IDI school model was to offer a framework for research but also to provide a research-based model for schools to reflect on, understand and improve their own practices to achieve sustainable pedagogical improvements with the help of digital technologies. There is a need for research-based, practice-oriented methods that help schools and teachers themselves reflect and investigate their own practices and thus improve them (Angelides et al. 2004). The aim of the present study was to examine how the model can be used to evaluate the existing practices of the schools used as examples and to make recommendations for improving the practices. The following research questions were constructed:

1. How does the innovative digital school model help to identify good practices and points for improvement in using digital technology for school change in the example schools?
2. How does the model reveal the essential differences in using digital technology for school change between the example schools?

## Methods

The study is an explanatory multiple case study for explaining how the theoretical model used supports the description of the cases and how the cases differed from each other (Yin 2014). The purpose is to increase understanding of the cases (Merriam 1998) and to create analytic generalisations for other cases and situations (Yin 2014). The study relies on holistic data collection strategies, following the mixed methods approach (Johnson and Onwuegbuzie 2004). The use of several approaches and methods leads to better understanding of the objects of investigation and mixing various methods gives a more accurate picture of what is going on, while different methods help to answer slightly different questions (Todd et al. 2004). They provide an opportunity to present a greater diversity of views (Teddle and Tashakkori 2003) and help us to understand complex phenomena (Newmann et al. 2000). In the study, the mixed methods followed the triangulation design model, the variant of multilevel research (Creswell and Plano Clark 2007) in which different methods are used to address different levels within the system (school) and the findings are merged into one overall interpretation.

### Context

Three basic education schools (grades 1–9) participated in the study. They all are located in the metropolitan area of Helsinki. The city’s education department is the local organiser of education and in principle; all schools have equal access to resources. The local administration organises the technical resources (network connections, computers and other digital tools, the virtual learning environment and other applications). The city has also provided good opportunities for in-service training about digital technology. However, the schools also have some capacity to acquire resources of their own choice, such as by participating in national development projects, or in voluntary teacher training events. All teachers have a university degree and they are qualified teachers.

All the schools are located in suburbs.

School A is located in a residential area of single-family houses. In the area, the unemployment rate was 4.8%, the proportion of inhabitants with a higher education background was 24.5% of the total, the number of inhabitants with a foreign background was 3.6% and the income per residence was €87,645 (Tikkanen and Selander 2014). The curriculum of the school emphasises environmental education and sustainable development. It is also the member of a programme which aims to reduce bullying. There were 360 pupils at school A in 2015.

School B is located in an area of small houses and blocks of flats. In the area, the unemployment rate was 7.1%, the number of inhabitants with a higher education background was 11.9%, the number of inhabitants with a foreign background was 9.9% and the income per residence was €57,335 (Tikkanen and Selander 2014). The school has no emphasis on any one subject; it aims to be a safe local school. There were 640 pupils at school B in 2015.

School C is located in an area of mainly blocks of flats. In the area, the unemployment rate was 15.1%, the number of inhabitants with a higher education background was 3.2%, the number of inhabitants with a foreign background was 23% and the income per residence was €32,182 (Tikkanen and Selander 2014). The school emphasises creativity and handicrafts, and it has two special classes emphasising digital technology from 3rd to 9th grade. The school has several special education classes, and it has organised preparatory teaching for immigrant pupils. There were 375 pupils in the school in 2015.

### Participants

Participants of the study were principals (one from each school), teachers with permanent positions and 9th grade pupils. Principals and teachers were not asked for their age; the mean of pupils’ age varied from 15.3 to 15.6 between schools. Table 2 shows the number of participants and their gender.

**Table 2:** The number of participants and their gender

	Principals	Teachers (survey) (f/m/not informed)	% of all teachers	Teachers (intensive study) (f/m)	Pupils (survey) (f/m)	% of all 9th grade pupils
School A	Male	10/6	53.3%	2/3	23/21	86.3%
School B	Male	13/9	47.8%	3/2	50/50	63.7%
School C	Female	12/5/2	61.3%	3/2	13/18	50.8%

The response rates to the survey of teachers and students at each school varied as described in Table 1. Schools and their principals, teachers and pupils participated voluntarily in the study. Permission to participate in the study was sought from parents of the pupils concerning surveys and the videotaping of lessons.

### Measures and data collection

From each school, the following data were collected:

#### Lesson observations

Five subject teachers using digital technology in teaching were recruited from each school for classroom observations and interviews. The lessons in which digital technology was somehow used by the teacher or pupils were chosen for observation. A pre-planned observation sheet of phenomena to be observed was used; the focus was on classroom practices, such as the nature of assignments, pupils’ activities in completing the assignments, the use of digital technology, pupils’ and teachers’ interaction regarding the assignment and technology as well as the focus of the teacher’s guidance. The teachers of the lessons that were observed were interviewed briefly before and after each lesson, concerning their observation about the goals and practices of the lesson. The lessons and the short interviews were videotaped; the videos were used to complement written observation notes. In Table 3 is a list of the lessons observed.

**Table 3:** The subjects and grade levels of the observed lessons

School	Subjects of the observed lessons (grade level)
A	English language (5), Mother tongue (Finnish) (9), Geography (7), History (8), Mathematics (9)
B	Computer science (8), English language (8), Mother tongue (Finnish) (8), Health science (8), Study Counselling (8)
C	English language (8), Mother tongue (Finnish) language (7), Mother tongue (Finnish literature) (8), Music (10), Religion (7)

#### Interviews

The principal and five teachers at each the school were interviewed using a semi-structured interview. The interviews focussed on the following themes: the use of digital technology in teaching, the school’s vision, the principal’s professional competence and its development, teachers’ collaboration practices and school community and the role of the principal. The principal was also asked about leadership issues. The interviews lasted about 1 h.

#### Surveys

Data about the use of digital technologies were collected through questionnaires from pupils and teachers. Both questionnaires were based on questionnaires developed in previous studies (Hakkarainen et al. 2000; Hakkarainen et al. 2001), and for this study, they were modified to take into account recent technological development (e.g. questions about the use of Internet were added).

The teacher questionnaire was sent to all teachers with permanent positions at the schools. It consisted of questions concerning the following topics:

1. Digital competence: 17 Likert-type statements (1 = not at all, 5 = very well); e.g. *How well do you manage spread sheet applications*, e.g. *Excel*
2. The use of digital technology: 41 statements concerning the use at school and at home, the use of various Internet services, the use of various digital applications with pupils (answer options were not at all—seldom—monthly—weekly—daily)
3. The need for support and training in using technology: Four Likert-type statements (1 = completely inadequate, 7 = completely adequate)
4. The usefulness of digital technology in some pedagogical practices: 20 Likert-type statements (1 = totally useless, 7 = totally useful); e.g. *Small*-*scale project works*, e.g. *information search for understanding a topic*.

The pupil questionnaire was sent to 9th grade pupils. The questionnaire consisted of questions concerning the following topics:

1. Digital competence: 17 Likert-type statements (1 = not at all, 5 = very well)
2. The use of digital technology: 33 statements concerning the use at school and at home, the use of various Internet services, the use of various digital applications at school (answer options were not at all—seldom—monthly—weekly—daily)
3. In which subjects is ICT used at school, also the frequency: Seven statements concerning school subjects (answer options were not at all—seldom—monthly—weekly—daily)

### Data analysis

Each type of data was first analysed separately as described below.

#### Lesson observations

Observation notes and related short interviews were used to categorise the pedagogical approach of each lesson. The classification was created by the researchers through abductive use of theory-informed and data-grounded analysis on the data (Timmermans and Tavory 2012). The pedagogical infrastructure framework (Lakkala and Ilomäki 2015) was applied to define the elements examined in the practices: technical structures (role and organisation of technology use), social structures (role and nature of collaboration), epistemic structures (practices of using and creating knowledge) and cognitive structures (cognitive challenge of tasks, support for pupils’ self-regulation and metaskills). Three categories were created for defining the prevailing pedagogical approach of each lesson:

1. *Structured content learning*: Technology was used for teacher presentations or structured practicing (e.g. drill-and-practice tasks), individual tasks, focus on learning factual and declarative knowledge, low cognitive challenge and no explicit attention to metacognitive aspects of working
2. *Learner*-*centred activating tasks*: Technology was used for information seeking or minor authoring tasks (e.g. short essays), mainly individual tasks but some sharing between pupils, small-scale knowledge production, mid-level cognitive challenge, but no explicit attention to metacognitive aspects of working
3. *Collaborative knowledge creation*: Versatile use of technical applications for knowledge creation (e.g. reports), working mainly based on pair or group work, open-ended task lasting more than one lesson, high cognitive challenge and modelling of working strategies

#### Interviews

The interviews were transcribed verbatim and then analysed following a theory-driven content analysis, using Atlas.ti software (version 7.1.5). The elements of the IDI school model (see Fig. 1) were used as categories to define which sections in each interview described which phenomenon of the school practices. The interview questions were designed to address the elements of the model, but in the analysis, we also considered that an answer referring to any of the elements might emerge under any question. In constructing the case descriptions of schools, the coding in Atlas.ti was used to extract all interview excerpts from an individual school concerning a certain school model element, in order to make the judgement and description of the nature and level of practices in that school.

#### Teacher and pupil surveys

The data were analysed with IBM SPSS 22. The means of items were compared using one-way ANOVA and Tamhane’s T2 post hoc tests.

#### Integration of the results from individual data sources

The dimensions and levels of each sub-element were constructed descriptively by combining the analysis results of separate data sets. The analysis was of iterative explanation building (Yin 2014): The analysis criteria, based on the IDI school model and described above, were first compared with the empirical evidence from the first case, and then revised and compared with the evidence from the other cases.

The dimensions of each phenomenon (elements of the IDI school model; see Fig. 1) and the data produced information about each element. Each element was scored in the following way: 1 (low level), 2 (average level) and 3 (high level). The scores were based on the analysis of all data sources, and the researchers together decided the scoring. In addition, the scores of the main elements were constructed as the means of the sub-elements. In Appendix, the analysis framework of the phenomena and the data is presented.

## Results

The results are first presented in the order of data and data analysis; the integration of the results is presented after that.

### Practices at each school, according to the interviews and classroom observations

The practices are presented following the order of the elements of the IDI School model in Table 3.

#### School A

Visions concerning digital technology related mainly to technical skills and resources. The visions were emerging; most teachers shared them, but the visions were not fully clear in teachers’ minds. The school had several common development projects going on and the importance of development activities was emphasised in the interviews.

Shared leadership came true in systematically organised teacher teams, which included all teachers, and the active role of the executive team. The principal’s networking included basic collaboration inside school and with municipal school administrators and parents. The principal acted as an enabler of teachers’ development efforts (e.g., organising resources for training), but also as the promoter of new development initiatives.

Teachers had various established collaboration practices, such as pedagogical workshops, co-teaching between teachers or sharing of teaching plans and materials through virtual forums. The school had multiple development practices, e.g. national and international projects, or periodic joint reflection of teaching. The teachers interviewed actively collaborated with colleagues at the same school, but they did not do much networking outside the school.

Teachers’ perceptions of digital technology in education focussed on aspects related to motivation, increased variability in methods or increased student-centredness and learning effectiveness, but there were few mentions about collaborative or creative activities. The usage of digital tools in teaching included a range of methods, from drill-and-practice tasks to challenging long-term project work. Three of the five lessons observed represented collaborative knowledge creation practices; some teachers appeared to use advanced pedagogical methods with digital technology.

Plans for developing common school-level practices, e.g. about media usage and study practices, had been started. A joint Media Week was organised annually. A virtual learning platform was established as an information channel for teachers, and its usage with pupils was actively promoted. Teachers’ experience of the school premises was that they were quite flexible, but some teachers mentioned the lack of a computer laboratory and the distribution of computers as problems. Pupils were involved in school-level activities in various ways; e.g. the pupils’ media team was responsible for documenting school events, and a training event in which pupils guided teachers to use social media had been organised. School-level networking was based on the activity of some teachers and their classes participated in national and international projects.

Most interviewees thought that too few computers were available for teaching, and that login in the laptops took too much time in lessons. The teachers had common plans about which digital skills to teach to pupils in each subject and grade. Digitally more-competent teachers had organised training sessions for less-competent colleagues about the central applications, and teachers were encouraged to participate in in-service courses organised by the city.

#### School B

Most of the teachers interviewed shared the opinion that there was no explicit vision in the school about digital technology. Some interviewees mentioned ensuring that pupils had good basic digital skills, whilst others emphasised the improvement in teachers’ digital competence, or flexible digital resources. Attitudes towards development efforts were positive, and some projects with other schools were going on, and there were plans for developing the school’s practices. However, the development interests appeared to be dependent on the motivation of individual teachers.

The teachers were divided into three administrative teams, each of which was allocated tasks based on needs; the teams had some responsibility of their own. The principal had established collaboration with the vice principals, the executive team and the principals of nearby schools, but there were no other explicit networks. The principal was described positively: the creator of a positive atmosphere, a pedagogical leader and a provider of resources for professional development.

Pedagogical collaboration included team discussions, some co-teaching practices, sharing of materials and informal discussions; it was mainly based on subject-specific groups and spontaneous and voluntary participation. The school had one common development programme (about learning to learn skills), but otherwise, development efforts included participation in training events and projects depended on the teachers’ own initiative. Two teachers mentioned an external organisation as a point of contact, but otherwise, networking included conventional partners: the city’s teacher training unit, teachers’ friends or parents. One teacher had no collaborators outside school.

Teachers’ pedagogical perceptions about digital technology included benefits concerning increased motivation, usage as a presentation tool, variation in methods and a useful writing tool. None of the teachers explicitly mentioned more challenging project- or inquiry-based methods or collaborative learning, but two of the lessons that were observed represented such practices.

Communication and sharing of materials among teachers was organised through web-applications, but otherwise no common knowledge practices were mentioned at the school, nor between teachers or pupils. Also, ICT courses for pupils were voluntary. Some teachers mentioned old-fashioned, inflexible premises and computer laboratories as a weakness; the problem was visible also in the lesson observations. One teacher had used older pupils as guides for younger pupils in technology use; otherwise, nobody described any practices for involving pupils in school-level activities. The interviews did not reveal any established school-level networks besides neighbouring schools participating in a common project.

Concerning the utility of digital resources, the teachers were not satisfied with the fixed computer laboratories and the shortage of equipment, especially mobile tools (like tablets). They were satisfied with the technical support but did not mention any examples of pedagogical support.

#### School C

Digital visions appeared not to be shared visions; the teachers interviewed mentioned basic digital skills, increasing technology use and more versatile practices, or explicitly said that they were unaware what the vision is. The experience of the atmosphere was as supportive of development efforts, and the school participated in various national and international projects.

Leadership was shared through subject-based and task-based teams, and some teachers had taken the responsibility for development projects. The principal had active collaboration with local institutions at various educational levels, and she had taken an active role in renewing common practices.

The teachers had many collaboration practices: working in teams or projects, informal discussions, sharing of ideas and materials and interdisciplinary co-teaching. The interviewees mentioned development practices such as projects and training sessions, but participation in them happened only occasionally and participation was voluntary, depending on the teacher. The teachers had networks with various stakeholders in institutions related to their subject, e.g. the church, music college or police.

Teachers’ perceptions about technology in education included conventional issues, such as individualised teaching, up-to-date information sources or useful tools for pupils’ work, but in general, teachers’ opinions were very positive. The pedagogical practices that were mentioned with technology were versatile but not very innovative, like individual knowledge production or rehearsal of content. None of the lessons that were observed included challenging collaborative knowledge creation activities.

The teachers had made common plans about the teaching of ICT and media communication to different grades of students, and web-applications were used for information sharing between teachers. Other common knowledge practices were not mentioned in the interviews. Some teachers experienced old, inflexible school premises as a challenge for advancing digitalisation, but a new room for project learning was under construction. Pupils’ involvement in school-level responsibilities and activities was not mentioned. The school had collaboration arrangements with external organisations through multiple national and international development projects.

The utility of technical resources was experienced as being at quite a good level, but the heterogeneity of teachers’ digital competence was mentioned as a challenge. Teachers had good opportunities to participate in courses organised by the city, and there had been some internal training events, but the emphasis had been on technical skills, not on pedagogical issues.

### Results of questionnaires with teachers and pupils

The results are presented in the order of the elements of the IDI school model shown in Table 3.

#### Results of the teacher questionnaire

##### Perceptions of using digital technology in education

Teachers were asked about the usefulness of digital technology in various pedagogical assignments. Table 4 shows the means, standard deviations (SDs) of teachers’ perceptions and the *p* value of statistical differences.

**Table 4:** Teachers’ perceptions of the usefulness of digital technology in various pedagogical assignments and statistical differences

	School A (*N* = 16)		School B (*N* = 21)		School C (*N* = 18)		*p* value
	Mean	SD	Mean	SD	Mean	SD	
Large projects	4.6	.892	5.4	1.958	5.2	1.581	
Small-scale project work	4.6	.730	6.0	1.203	6.1	.938	A < B, C, .000
Students’ independent work	4.1	.998	5.3	1.354	5.1	1.349	
Students’ inquiry work	4.1	.957	5.5	1.327	5.2	1.517	
Students’ fieldwork	3.4	.892	4.7	2.153	4.9	1.697	
Virtual laboratory work and simulations	3.3	1.014	4.6	2.224	4.6	1.688	
Practicing skills and methods	4.0	.966	4.9	1.513	6.0	1.138	A < C, .000
Small-scale product	4.3	.704	5.3	1.189	6.1	1.056	A < C, .000
Discussion on the net	3.4	1.094	4.8	1.692	5.2	1.505	A < C, .005
Presenting information and support for illustration	4.4	.892	5.7	1.426	6.32	.907	A < B,.009 A < C .000

There were statistically significant differences in the following perceptions of the usefulness of digital technology: At school A, teachers’ evaluation scores were statistically significantly lower than the scores of teachers at the other schools in the following pedagogical practices: small-scale project work *F*(2,54) = 12.841, *p* = .000; practicing skills *F*(2,54) = 10,866, *p* = .000; small-scale products (like writings during one lesson) *F*(2,54) = 12.256, *p* = .000; net discussions related to the topic *F*(2,54) = 6.412, *p* = .003; and presenting information and support for illustration *F*(2,54) = 12.148, *p* = .000. Tamhane’s T2 post-hoc comparisons were used to calculate the differences between the schools.

##### Pedagogical practices with digital technology

Teachers were asked about the use of various digital applications and Internet services in their own teaching; there were no statistically significant differences between schools in how much they reported using various applications and the Internet.

Teachers were also asked about using digital technology in various pedagogical practices. In Table 5, the means and SDs of all practices are presented. There were a few statistically significant differences in the reported use of digital technology.

**Table 5:** The means and SDs of pedagogical practices with digital technology and statistical differences

	School A (*N* = 16)		School B (*N* = 21)		School C (*N* = 18)		*p* value
	Mean	SD	Mean	SD	Mean	SD	
Large projects	3.3	1.483	3.6	2.376	4.1	1.731	
Small-scale projects	3.6	.256	4.8	.284	5.6	.231	A < C, .000
Students’ independent work	2.1	1.289	2.6	1.359	2.8	1.689	
Students’ inquiry work	2.6	1.408	2.9	1.590	3.3	1.638	
Students’ fieldwork	2.0	1.033	2.1	1.315	3.0	1.534	
Virtual laboratory work and simulations	1.6	.957	1.6	1.284	2.2	1.200	
Practicing skills	2.7	1.352	3.7	1.683	5.2	1.581	A < C, .000
Small-scale products	3.6	.964	4.6	1.499	5.4	1.145	A < C, .000
Discussion on the net	1.7	.873	2.6	1.690	2.8	1.555	
Presenting information and support for illustration	3.5	1.414	4.6	1.962	5.4	1.243	A < C, .001

The statistically significant differences were found in the following items: small-scale projects *F*(2,54) = 13.233, practicing skills, *F*(2,54) = 10.988, *p* = .000; small-scale products (like writings) *F*(2,54) = 9.084, *p* = .000; and information presenting and support for illustration *F*(2,54) = 5.934, *p* = .005. Tamhane’s T2 post-hoc comparisons were used for calculating the differences between the schools.

##### Teachers’ digital competence

The results showed, first, that there were no statistically significant differences between schools in teachers’ self-evaluated digital competence, and that teachers evaluated their competence in basic digital application as being quite high (scale 1–5), such as using email (mean 4.7), searching for information on the Internet (mean 4.7), word processing (mean 4.4), loading files from the Internet (mean 4.2) and using the digital learning environment (mean 3.8). These formed a group of basic digital competence. The second group of applications were using spreadsheets (mean 3.2), digital image processing (mean 3.1), graphics (mean 2.9) and social forums (mean 2.9). The lowest means were in virtual meeting tools (mean 2.3), creating www-pages (mean 2.3), publishing tools (mean 2.2), writing a blog (mean 2.2), publishing www-pages (mean 2.0), producing information to wiki (mean 1.9), voice and music (mean 1.9) and programming (mean 1.4).

##### Pedagogical and technological training and support

**Fig. 2:**
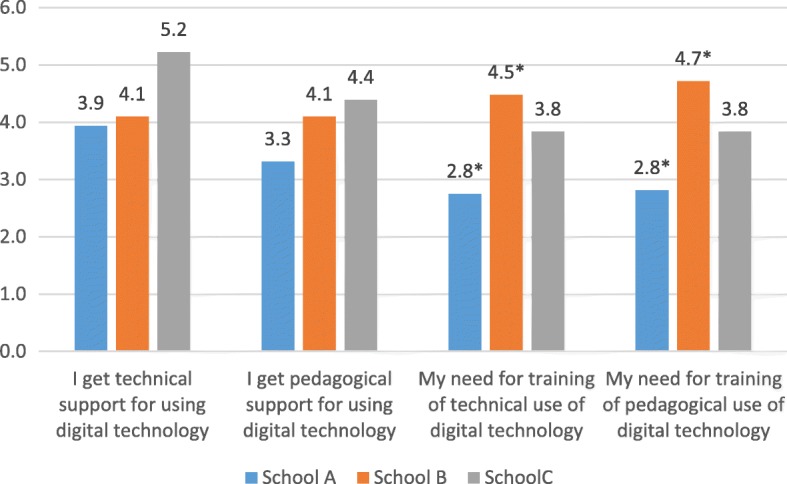
Teachers’ need for support and training of digital technology

Figure 2 shows the means of teachers’ need for support and training for using digital technology.

The evaluation of teachers at school A was that they needed both technical and pedagogical training less than teachers at the two other schools, and there was a statistically significant difference between schools A and B in need for technical training: *F*(2,54) = 9.993, *p* = .000; and in need for pedagogical training: *F*(2,54) = 12.719, *p* = .000, indicated with * in Fig. 2.

#### Results of the pupil questionnaire

##### Pedagogical practices with digital technology

Pupils were asked which applications they use at school. In Table 6, the means and SDs of those applications in which there were statistically significant differences between the schools are described.

**Table 6:** Means, SDs and statistical differences of digital applications and pedagogical practices used

	School A (*N* = 44)		School B (*N* = 100)		School C (*N* = 31)		*p* value
	Mean	SD	Mean	SD	Mean	SD	
Using digital applications							
Using word processing	3.7	.544	2.8	.857	3.0	1.000	A > B,.000; A > C, .002
Using spreadsheets	2.7	.694	1.8	.899	2.1	1.076	A > B, .000
Using email	4.0	.590	2.4	1.066	2.8	1.098	A > B, C, .000
Information search from the Internet	4.0	.549	2.9	.993	3.3	.945	A > B, .000¸ A > C, .002
Publishing on the Internet	2.5	1.045	1.9	.968	2.5	1.434	A > B, .002
Using social forums	3.0	1.562	2.3	1.228	3.3	1.137	C > B, .000
Using learning environments	3.5	.952	2.6	.998	2.3	.973	A > B, C, .000
Publishing in a web blog	3.0	1.137	1.6	.960	2.2	1.267	A > B, .000
Publishing pictures, texts or reports	2.5	1.000	1.9	.988	1.9	1.221	A > B, .002
Pedagogical practices with digital technology							
Developing my thoughts about the topic in a collaborative discussion	2.7	.851	1.7	.886	2.0	1.251	A > B, .000
Teacher guidance through the net for independent learning	2.4	.868	1.7	.949	2.5	1.312	A > B, .001
Freedom to surf in the Internet when assignments are done	3.4	1.203	2.7	.973	3.7	.965	A > B, .004, B < C, .000
Contact with pupils in other schools via email or the Internet	3.0	1.285	2.2	1.242	2.8	1.440	A > B, .001
Information search from the Internet	3.9	.443	2.9	.865	3.5	.890	A > B, .000 B < C, .004
Publish pictures, texts of reports	2.5	1.000	1.9	.988	1.9	1.221	A > B, .003

The statistical significance of differences in means between the pupils of schools was analysed by using one-way ANOVA. The analysis indicated statistically significant differences in the means in the following items: using word processing: *F*(2,172) = 18.909, *p* = .000; using spreadsheets: *F*(2,172) = 16.686, *p* = .000; using email: *F*(2,172) = 38.490, *p* = .000; using social forums: *F*(2,172) = 9.940, *p* = .000; publishing in a web blog: *F*(2,172) = 22.253, *p* = .000; using learning environments: *F*(2,172) = 17.316, *p* = .000; publish pictures, texts or reports: *F*(2,172) = 5.811, *p* = .004; develop my thoughts about the topic in a collaborative discussion: *F*(2,172) = 14.735, *p* = .000; teacher guidance through the net for independent learning: *F*(2,172) = 9.678, *p* = .000; freedom to surf in the Internet when assignments are done: *F*(2,172) = 15.361, *p* = .000; and contact with pupils in other schools via email or the Internet: *F*(2,172) = 8.367, *p* = .000; information search from the Internet: *F*(2,172) = 22.464, *p* = .000; publishing in the Internet: *F*(2,172) = 7.281, *p* = .001. Tamhane’s T2 post-hoc comparisons were used for calculating the differences between the schools.

There was also a difference in the statement about the use of ICT during leisure time for school work, in which pupils at school A had higher scores than pupils at the other schools. The statistically significant differences were between school A (*M* = 3.7, SD = .553) and schools B (*M* = 2.3, SD = .833) and C (*M* = 2.2, SD .956) (*F*(2,172) = 55.259, *p* = .000).

##### Pupils’ digital competence

Pupils at all three schools liked to use ICT at school, and there were no statistically significant differences concerning the statements measuring this: the use of ICT is easy (*M* = 4.2, SD = 1.034), the use of ICT makes learning more interesting (*M* = 3.9, SD = 1.111) and pupils would like to use ICT more at school (*M* = 3.8, SD = 1.192). Furthermore, there were no statistically significant differences in the use of technology at home and during leisure time.

Pupils also evaluated their competence in using various digital applications. The statistically significant differences in means and SDs between the pupils from the three schools are described in Table 7.

**Table 7:** Pupils’ self-evaluated digital competence in some applications (means, SDs and statistical differences)

	School A (*N* = 44)		School B (*N* = 100)		School C (*N* = 31)		*p* value
	Mean	SD	Mean	SD	Mean	SD	
Word processing	4.5	.504	4.0	.953	3.5	.926	A > B, .000 A > C, .000
Spreadsheets	4.0	.731	3.0	1.303	2.9	.806	A > B, .000 A > C, .000
Email	4.9	.321	4.6	.680	4.2	1.036	A > C, .000
Writing in web blog	3.9	.830	2.9	1.463	3.0	1.390	A > B, .000
Virtual learning environment	4.4	.542	3.8	1.170	3.4	1.174	A > B, .000 A > C, .000

The differences were analysed by using one-way ANOVA. No differences were found in applications which tend to be less used in schools, such as digital image processing, publishing tools, voice and music applications or programming. The analysis indicated statistically significant differences in means between pupils of participating schools in the following items: word processing *F*(2,172) = 13.287, *p* = .000; spreadsheets *F*(2,172) = 15.092, *p* = .000; email *F*(2,172) = 10.002, *p* = .000; information search from the Internet *F*(2,172) = 6.492, *p* = .002; writing a web blog, *F*(2,172) = 9.441, *p* = .000; and using virtual learning environments *F*(2,172) = 9.042, *p* = .000. Tamhane’s T2 post-hoc comparisons were used for calculating the differences between the schools.

### Overview of the level of practices in the schools

In Table 8, the results of the separate data sets have been integrated and scored for each school.

**Table 8:** Evaluated level of practices in each school

Phenomenon investigated	School A	School B	School C
A. Vision of the school	2.3	1.3	2.3
A1. The vision of using digital technology	2	1	2
A2. Consensus about the vision	2	1	2
A3. Intentional development-orientation	3	2	3
B. Leadership	2.7	2.0	3.0
B1. Shared leadership	3	2	3
B2. Networking of the principal	2	1	3
B3. Role of the principal	3	3	3
C. Practices of teaching community	3.0	1.7	2.7
C1. Pedagogical collaboration and sharing of expertise	3	1	3
C2. Development practices	3	2	2
C3. Networking of teachers	3	2	3
D. Pedagogical practices	2.5	1.5	2.0
D1. Perceptions of using digital technology in education	2	2	2
D2. Pedagogical practices with digital technology	3	1	2
E. School-level knowledge practices	2.5	1.0	2.0
E1. Common knowledge practices with technology	3	1	2
E2. Physical premises	2	1	2
E3. Students’ involvement in school level activities	3	1	1
E4. School-level networking	2	1	3
F. Digital resources	2.75	1.75	2.0
F1. Utility of technical resources	3	1	2
F2. Pupils’ digital competence	3	2	2
F3. Teachers’ digital competence	2	2	2
F4. Pedagogical and technical support	3	2	2

The scores show differences between schools: schools A and C are ‘strong’ schools in several major elements. At school A, digital resources are at an especially high level, and in general, school-level working practices are at a high level. At school C, leadership practices and teaching community practices are at a high level. School B has the lowest scores in every major element. In the ‘Discussion’ section, we will discuss about the differences more in detail.

## Discussion

In the study, we investigated the practices at three schools based on six elements defined in the innovative digital school model. We aimed to find out, first, if those elements could help in defining good practices and suggestions for improvement for developing the schools with digital technology; and second, if the model revealed essential differences between the schools.

### Good practices and points for improvement in the example schools

In order to answer the first research question about how the IDI school model helps to identify good practices and points to be improved in using digital technology for school change, we describe the practices of each school separately.

Among the characteristics of school A were advanced and established practices in shared leadership, practices of the teaching community, advanced pedagogical practices with technology and school-level knowledge practices, including involvement of pupils and systematic promotion of their digital competence through pedagogical activities. However, shared visions about digital technology were only emerging, teachers’ digital competence was only average and the perceptions in the pedagogical usage of technology had considerable variety between teachers, although there were examples of inspiring pedagogical methods. Teachers did not report needing support for using technology which probably indicates both quite a good level of digital competence and well-organised support practices in the school. Pupils’ self-reported digital competence was at a high level especially concerning basic applications. Pupils reported using technology quite often during leisure time for school-related activities, and at school for various basic activities, but also for collaboration and networking. Based on the results, the following suggestions for improvements can be made for school A: (1) the teaching staff should focus on crystallising and sharing the school’s visions in using digital technology as the basis for further development (elements A1 and A2); (2) teachers should share their pedagogical ideas and experiments, e.g. in organised meetings and workshops (elements C1 and C2); and (3) teachers should develop their digital competence, such as by making use of the training resources made available by the city and by organising school-level small-scale training (elements F2 and F4).

School B had some shared leadership practices and the principal was appreciated, but otherwise the school was not very advanced in any of the measures. Attitudes towards development efforts were positive, but established practices were lacking. There were teachers who collaborated with each other, participated in development projects and used digital technology in teaching in advanced ways, but activity was based on teachers’ own initiative and voluntariness. Especially at the school level, knowledge practices were minimal, both concerning the promotion of pupils’ involvement and digital competence, and school-level networking. Teachers at the school reported needing both technical and pedagogical support in using digital technology. For school B, based on the results, the following suggestions for improvements can be made: (1) it is important to create a common vision for developing the use of digital technology (element A1) and promote development orientation among teachers (element A3). (2) The principal and the management team should create and organise systematic common practices to carry out improvements in all developmental areas (elements in C). (3) The digital resources should be evaluated and developed (all elements in F) and especially teachers’ digital competence should be improved (elements F3 and F4).

School C represents a school with high-level leadership practices, and a strong collaboration culture both inside the school and in the active external networking of both the principal, teachers and the whole school. The school had a strong development orientation in general, but it had not yet become true in the school-level knowledge practices, digital resources or advanced practices of using technology in teaching. School C has much potential for improvement, and based on the results, the following suggestions for improvements can be made: (1) the usage of digital technology for school improvement should be more deliberate through agreements of shared visions (elements A1 and A2); (2) the school should create systematic development of pedagogical and knowledge practices (elements D and E); and (3) all pupils’ and teachers’ digital competence should be improved, both with pedagogical practices (element D2) and training and support (elements F2, F3 and F4).

### Differences between the schools investigated

To answer the second research question about how the model reveals essential differences in digital technology for school change, we compared the practices of schools by summarising the results of data analyses.

The results of the study indicate that there were some clear differences between the schools, although they also had a lot in common, especially in the principal’s role and teachers’ digital competence; common characteristics might be a result of common policies and practices of the city in these issues. Such elements, which are strongly dependent on school-level decisions, differed between the schools. Included here are teachers’ pedagogical practices and school community’s practices, including sharing of vision-level decisions. According to previous studies (Vieluf et al. 2012; OECD 2014), shared community-level practices are central to sustainable school improvement, but currently they represent practices which are not yet widespread in schools and require extending the teachers’ professional role beyond only taking responsibility for their own teaching in classrooms.

A clear difference between the three schools was in the presence or absence of practices involving pupils in school-level activities. Only at school A had shared, established practices for pupil engagement at school-level been developed, such as responsible pupil teams (e.g. media and environment teams) or pupils as guides in using digital technology. Various participatory practices presume seeing pupils in an active role in the classroom or at school, not only as objects of teaching during lessons (Facer 2012; Kehoe 2015; Pereira et al. 2014).

Also, the nature of pedagogical practices with digital technology differed between schools. At school A, pupils reported using digital technology more than pupils at the other two schools, both in the classroom and at home for school-related activities. The use focused on general applications and pedagogically ‘advanced’ practices, such as using a virtual learning environment and collaborating via the web. These practices probably helped to improve pupils’ basic digital competence: the regular use of digital tools was an essential condition for competence learning (see also OECD 2011; Aesaert et al. 2015). Furthermore, classroom practices were most advanced at school A and a comparisons of the teachers’ survey answers between the schools indicated that teachers at school A used and believed less in teacher-centred practices with digital technology than teachers at school C.

**Fig. 3:**
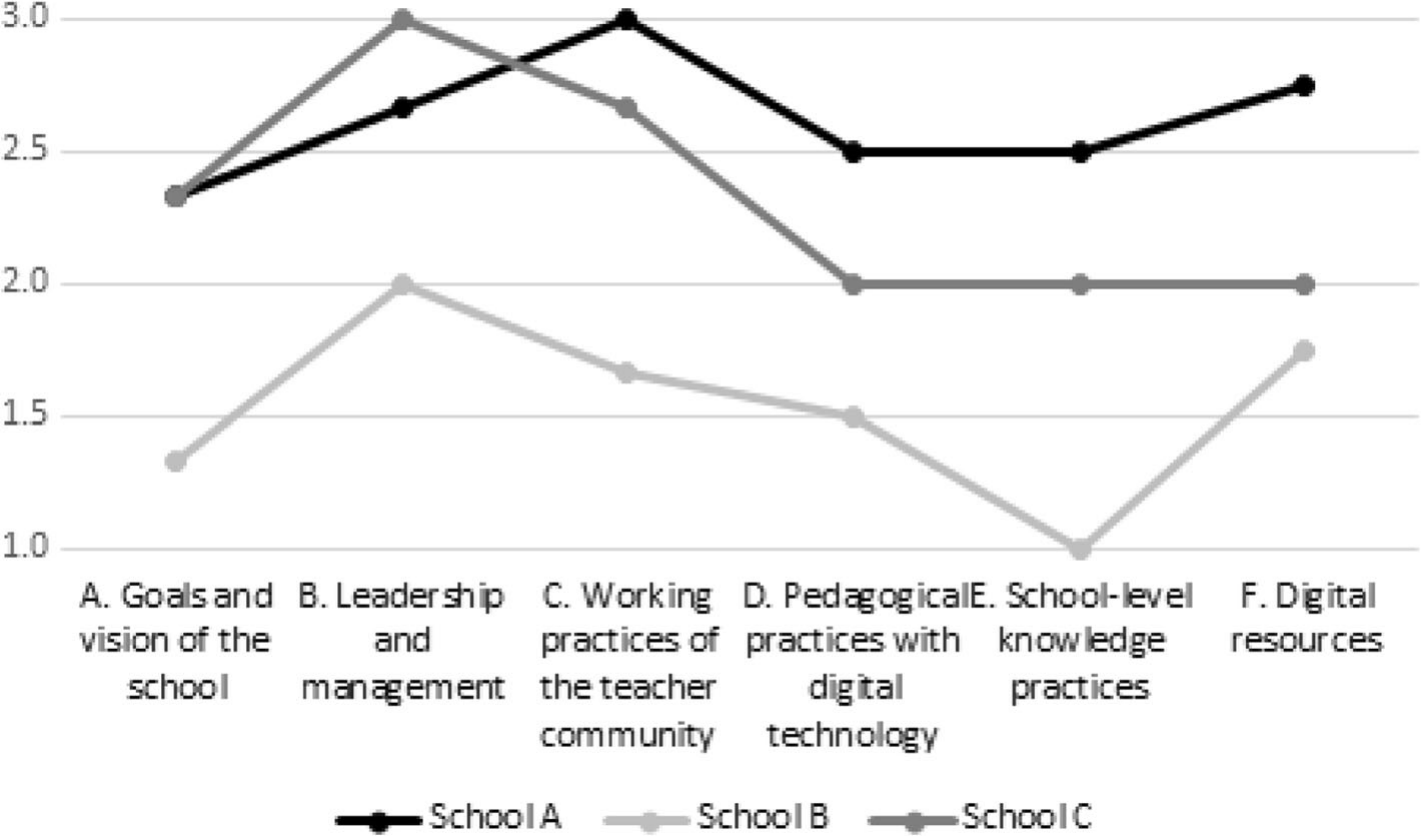
A summary of the scores of the three schools in the elements of the IDI school model

The innovative digital school model was not developed primarily for detailed comparisons of differences between schools. A more useful approach is to examine school profiles: the shape of the profile demonstrates the emphasis on the practices inside a school, and the level of the profile elements helps each school to position its strengths and development needs compared with reference schools. Figure 3 presents the results of Table 9 in a visual form illustrating the profiles of the three schools investigated.

The profiles demonstrate the differences between the schools: school A has quite advanced practices in all elements; school C is high in school-level practices involving teachers and the principal, but only average in practices directly affecting pupils; and school B is least-developed in all elements, but highest-developed in leadership and digital resources. We propose that one reason for the differences between schools is the level of vision and how well it is shared among teacher community. Schools A and C had remarkably higher scores in the elements of goals and the vision compared with school B (although even schools A and C could improve on this). These results are in line with previous research according to which an explicated and shared vision is a key element in school improvement and change (see, e.g. Senge et al. 1994; Antinluoma et al. 2018). At school B, the vision and goals, pedagogical practices with digital technology and school-level knowledge practices were all at a low level, although the digital resources are almost the same as at school C. For benefitting from digital technology in improving pedagogy, collaborative visions and efforts especially focusing on that are needed (Laurillard 2008); technology does not change pedagogical practices per se, which describes the situation at school B. At both schools A and C, the elements related to vision, leadership and teacher community received good or even high scores, but school A was more advanced in pedagogical practices with technology. It seems that to develop high-level pedagogical practices with technology, deliberate effort is needed.

## Conclusions

### Validity of the innovative digital school model

The two aims of the IDI school model, to reveal good practices and points for development, as well as to expose differences, were fulfilled, from which we interpret that *analytic generalisation* (Yin 2014) from the model is possible. With qualitative data (classroom observations and interviews), we were able to identify new and innovative practices in the school context, developed in the schools for their individual needs. The quantitative data supported the findings based on qualitative data. Innovative practices were found, especially at the school which was evaluated as being the most advanced in all elements. One of the schools was least-developed in all the measures investigated, and the third school was in between: it had a strong development culture generally, but the focus of the development work had not been on using digital technology as a vehicle for change. In the latter two schools, digital technology was taken into use by individual teachers and often without integrating pedagogy and technology.

The IDI school model as a framework for investigating differences worked particularly well for those elements which are mainly the responsibility for leadership inside a school (visions of the school, practices of teaching community and school-level knowledge practices); there were clear differences in these between the schools, especially according to the qualitative data. The three schools had differences even though they each follow the same curriculum, and the same detailed legislation. The teachers’ educational background is homogeneous, and the schools are located in the same city, which is responsible for providing the resources for all the city’s schools. The role of the city probably explains why there were no statistically significant differences between teachers’ self-estimated digital competence and the use of digital technology in general.

Results of the qualitative and quantitative data were somewhat contradictory in the use of digital technology in classrooms. In the teacher surveys, there were no statistically significant differences between schools, but there were in the pupil surveys. Our explanation is that pupils use technology in some lessons so much that it affects the overall experience, and that pupils in 9th grade use technology more than pupils in lower grades.

Another contradictory issue in the surveys was the result of pedagogical practices. The teachers participating in the observations and interviews were probably more interested in digital technology and their practices were more advanced than the practices reported in the survey by many more teachers. As Kivinen et al. (2016) suggested, the technology use of the majority of teachers might represent the use of technology per se, which leads to a pragmatic solution in which technology does not support a knowledge creation approach in learning but is used for practical experiments and learner-centred activities.

The schools that were examined are located in areas of different socioeconomic backgrounds. The results do not show differences based on the background, which probably indicates the homogeneity of Finnish schools. All schools receive the same resources from the city, and parents do not make financial contributions for the education. The school from the area of lowest socioeconomic status has participated in various projects during years, and this has promoted the capacity of the teaching staff. Teachers’ development orientation has supported the school to develop advanced practices regardless of challenging socioeconomic background of the pupils.

The results of the study proved that mixed methods are needed when investigating the practices of a whole school. Using only the survey data would not have revealed some of the central differences between the schools and would have given a quite narrow view of the situation at each school. For the qualitative data, it would not have informed about the use of digital technology and the competence in using it. Collecting qualitative data requires more resources than using only surveys. However, we experienced that our data collection model (five teacher interviews and lesson observations, a principal interview and a survey of teachers and highest grade of pupils) was a reasonably inexpensive and valid way to examine the practices of a school.

### Practical implications

The IDI school model is an attempt to address the need for practice-oriented methods that help schools and teachers to reflect on their own practices and improve them (Angelides et al. 2004), and to narrow the gap between empirical research and practical school work (Wikeley et al. 2005), especially related to the change processes of implementing new digital technologies in education.

The IDI school model can be used in schools as a shared conceptual framework for collective reflection, discussion and strategy planning. We have already had some promising experiences about using it in the in-service training of teachers and principals. The model can also be applied to collect best-practice examples from different schools and disseminate them to other schools, or to make school visits and benchmarking of practices more systematic.

At the municipal and national level, educational administrators may have an interest in evaluating the status of using digital technology in schools. As our study witnessed, quantitative data have limitations in describing collaborative pedagogical and working practices. Qualitative methods are important, but there is a need for accessible methods for collecting data widely about the current state of art in schools. The methods, experiences and results of the present study can work as a starting point for developing scalable methods.

As a policy-level implication, we suggest that local and national school administration focus on schools as knowledge work organisations when aiming to improvements, such as to increase the quality of pedagogical and knowledge practices with digital technology in schools. We suggest that all elements of the innovative digital school model be considered, and that the start should be committing the staff to change, by creating shared visions and aims about pedagogical development through digital technology, and by supporting school-level practices including both pupils and teachers.

### Future research

In the present study, we used data from three schools to examine the applicability and validity of the IDI school model for evaluating the development of schools through digital technology. All three schools were in the same city and had similar municipal resources for digital technology and in-service teacher training, which allowed differences to be revealed, especially in those practices that schools can influence individually in that context. In future research, it would be important to test the model with a larger collection of schools from different contexts (size, location, socioeconomic background, etc.) and from different countries and cultures, thus also confirming the validation of the model.

Another interesting line of research would be to conduct studies in which the development of the same schools was followed longitudinally. Such studies could include interventional aspects: the investigated schools would get feedback and support from researchers to develop their practices further, and new data would be collected after some period for evaluating the influence of deliberate development efforts.

In the future, schools will face even more challenges and requirements that the school community will have to answer. The best and most effective schools reflect their practices and constantly improve their ways of working. We believe that the innovative digital school model offers a tool for schools and for researchers involved in this work.

## Appendix

**Table 9:** The analysis framework of the phenomena and the data

Investigated phenomenon	Dimensions of the phenomenon	Data sources^a^
A. Visions of the school		
A1. Visions of using digital technology	1. No clear visions 2. Emphasis on technical issues, like increasing equipment 3. Using digital technology for overall improvement	Teacher interviews, principal interview
A2. Consensus about the vision	1. No common vision 2. Emerging; vision not present in daily work 3. Consensus of the vision; the vision is important for the school	Teacher interviews, principal interview
A3. Intentional development orientation	1. No emphasis on development efforts 2. Individual initiatives supported, positive attitudes towards change 3. Focused collaborative development practices, the whole community accepts and participates	Teacher interviews, principal interview
B. Leadership		
B1. Shared leadership	1. Principal-centred community, no teams 2. Occasional teams or teams based on voluntary participation 3. Commonly agreed teacher teams, true responsibilities	Principal interview, teacher interviews
B2. Networking of the principal	1. No networking or only for administration 2. Networking with colleagues and administration, mainly with the same educational level 3. Active networking with various kinds of educational institutions and actors outside educational field	Principal interview
B3. Role of the principal	1. Mainly routine management 2. Good human resources leader, positive for development but not proactive 3. Organiser, developer of resources, initiator of improvement	Teacher interviews, principal interview
C. Practices of the teaching community		
C1. Pedagogical collaboration and sharing of expertise	1. Occasional collaboration between teachers of same subjects or class levels; material shared between a few teachers 2 Collaboration between teachers of same subjects or class levels; experiences shared occasionally in the school 3. Organised pedagogical collaboration and sharing practices	Teacher interviews, principal interview
C2. Development practices	1. No collaborative development practices 2. Occasional development activities based on active individuals; freedom to develop 3. Established collaborative and individual development practices	Teacher interviews, principal interview
C3. Networking of teachers	1. No networking or few teachers are networking 2. Several teachers have networks, but mainly with colleagues of the same subject 3. Several teachers active in networks, various types of contacts inside and outside school	Teacher interviews, teacher questionnaires
D. Pedagogical practices		
D1. Perceptions of using digital technology in education	1. Technology replacing teacher’s routines or for small-scale content learning 2. Technology as pupils’ tool for preparing and presenting pieces of work and for information search; emphasis on individual learning 3. Technology for diverse collaborative and creative learning activities	Teacher questionnaires, teacher interviews
D2. Pedagogical practices with digital technology	1. Technology used in a teacher-centred way, content learning activities, applications related to textbooks or teacher presentations 2. Technology used according to the teacher; learner-centred activating tasks in individual lessons, short (one or two lessons) individual or small group activities, teacher-directed assignments 3. Teachers use technology in multiple ways; process-type activities and integrated projects; technology as a tool, but also used to improve digital competence	Classroom observations, teacher interviews, teacher and pupil questionnaires
E. School-level knowledge practices		
E1. Common knowledge practices with technology	1. No or limited common practices 2. Some shared practices or agreements, concern mainly technology 3. Agreements, models and guidelines related to various knowledge practices and competencies	Teacher interviews, principal interview, classroom observations
E2. Physical premises	1. Inflexible spaces mainly for class teaching 2. Various types of spaces, but not enough flexibility and possibilities 3. Premises planned according to versatile pedagogical needs	Teacher interviews, classroom observations, principal interview
E3. Pupils’ involvement in school level activities	1. No involvement other than the traditional pupil’s role 2. Occasional and emerging activation of pupils 3. Several and various types of pupils’ involvement and responsibilities	Teacher interviews, classroom observations, principal interview
E4. School-level networking	1. No networking 2. Some networking, related to specific issues or individual teachers 3. Systematic, established contacts and collaboration partners	Teacher interviews, principal interview
F. Digital resources		
F1. Utility of technical resources	1. Centralised, insufficient resources, not working properly 2. Resources decentralised but insufficient 3. Good resources, technology decentralised to various spaces, various types of equipment	Teacher interviews, classroom observations, principal interview
F2. Pupils’ digital competence	1. Pupils’ digital competence based on informal learning outside school; no plans or activities to support it 2. Pupils’ digital competence supported by a specific course or some individual teachers; not provided for all pupils 3. Digital competence is systematically supported; strategies about teaching digital skills in various subjects and grade levels	Pupil questionnaires, teacher interviews, classroom observations
F3. Teachers’ digital competence	1. Digital competence varies, competence improvement based on individual decisions, no common lines, focus on technical skills 2. All teachers have basic competence, focus on technical skills 3. All teachers have multiple types of digital competence, but the level varies; focus on the pedagogical use of technology	Teacher questionnaires, teacher interviews, principal interview, classroom observations
F4. Pedagogical and technical training and support	1. Some teachers responsible for technical support, no organised pedagogical support 2. Support organised but not sufficiently; focus on technological support 3. Well-organised support; in technical problems help easily available; also pedagogical support available	Teacher interviews, teacher questionnaires, principal interview

^a^The data sources are listed in the order of importance
